# Profile of tunisian students consulting in psychopedagogy unit

**DOI:** 10.1192/j.eurpsy.2021.1054

**Published:** 2021-08-13

**Authors:** N. Messedi, N. Abdelkafi, O. Bouattour, A. Chamseddine, A. Ghorbel

**Affiliations:** 1 Psychiatry (b), Hedi Chaker University hospital, sfax, Tunisia; 2 Class Toxicology Unit, hygiene Loboratory, sfax, Tunisia; 3 Psychiatry, Hedi Chaker University hospital, sfax, Tunisia

**Keywords:** mental disorder, Students, academic result, mental disorder, antidepressor, students, academic result

## Abstract

**Introduction:**

A signifiant proportion of adolescents and young adults suffer from mental disorders that interfere with their development and influence their academic and professional succes.

**Objectives:**

To describe the soscio-demographic and clinical profile of tunisian students who have consulted in the psycho-pedagogy unit.

**Methods:**

A retrospective descriptive study, data were collected from the files of 359 pupils and students having consulted in the psycho-pedagogy unit of the psychiatry (B) department of the Hedi Chaker university hospital of Sfax in Tunisia, from 2014 to 2018.

**Results:**

The average age of students was 20.38 ±2.38 years old. The Sex ratio = 0.95 (175 M / 184 W). Students were undergraduates in 55.4 of the cases. THey are smokers in 19% and consume alcohol in 7% of case. A family psychiatric history was present in 34% of students and personal history in 25%. A difficult childhood was found in77.5% of patients and their academic results were low (< 10/20) in 54%. Adjustement disorders were the most frequent disorders in psycho-educationel counseling (47% of case). The most prescribed psychotropic drugs were antidepressants (31.8%) followed by anxiolytics (23.7%) and the withdrawel syndrome was found in 18% of patients.

**Conclusions:**

A students with a difficult childhood, low academic results and adjustement disorders such is the profile most frequently encountered among patients consulting the psychopedagogy unit. In this way,the promotion and protection of adolescent’s health particulary pupils and students benefits not only for their own health, but also for the economic and the society.

